# Mindfulness-based online intervention on mental health and quality of life among COVID-19 patients in China: an intervention design

**DOI:** 10.1186/s40249-021-00836-1

**Published:** 2021-05-17

**Authors:** Ming-Yu Si, Wei-Jun Xiao, Chen Pan, Hao Wang, Yi-Man Huang, Jun Lian, Winnie W. S. Mak, Zhi-Wei Leng, Xiao-You Su, Qiu-Ping Tang, Yu Jiang, Lu-Zhao Feng, Wei-Zhong Yang, Chen Wang

**Affiliations:** 1 School of Population Medicine and Public Health, Chinese Academy of Medical Sciences and Peking Union Medical College, 9 DongDanSanTiao, Dongcheng, Beijing, China; 2 Department of Clinical Psychology, The Third Xiangya Hospital of Central South University, No. 138 Tongzipo Road, Yuelu, Changsha, Hunan China; 3 Diversity and Well-Being Laboratory, Department of Psychology, The Chinese University of Hong Kong, Shatin, NT, Hong Kong, China; 4 Chinese Academy of Medical Sciences and Peking Union Medical College, Beijing, China; 5 National Clinical Research Center for Respiratory Diseases, Beijing, China

**Keywords:** COVID-19, Internet, Mindfulness-based intervention, Mental health, Randomized controlled trial, China

## Abstract

**Background:**

COVID-19 can lead to increased psychological symptoms such as post-traumatic stress disorder (PTSD), depression, and anxiety among patients with COVID-19. Based on the previous mindfulness-based interventions proved to be effective, this protocol reports a design of a randomized controlled trial aiming to explore the efficacy and possible mechanism of a mindful living with challenge (MLWC) intervention developed for COVID-19 survivors in alleviating their psychological problems caused by both the disease and the pandemic.

**Methods:**

In April 2021, more than 1600 eligible participants from Hubei Province of China will be assigned 1:1 to an online MLWC intervention group or a waitlist control group. All participants will be asked to complete online questionnaires at baseline, post-program, and 3-month follow-up. The differences of mental health status (e.g. PTSD) and physical symptoms including fatigue and sleeplessness between the COVID-19 survivors who receiving the online MLWC intervention and the control group will be assessed. In addition, the possible mediators and moderators of the link between the MLWC intervention and target outcomes will be evaluated by related verified scales, such as the Five Facets Mindfulness Questionnaire. Data will be analyzed based on an intention-to-treat approach, and SPSS software will be used to perform statistical analysis.

**Discussion:**

The efficacy and potential mechanism of MLWC intervention in improving the quality of life and psychological status of COVID-19 survivors in China are expected to be reported. Findings from this study will shed light on a novel and feasible model in improving the psychological well-being of people during such public health emergencies.

*Trial registration* Chinese Clinical Trial Registry (ChiCTR), ChiCTR2000037524; Registered on August 29, 2020, http://www.chictr.org.cn/showproj.aspx?proj=60034.
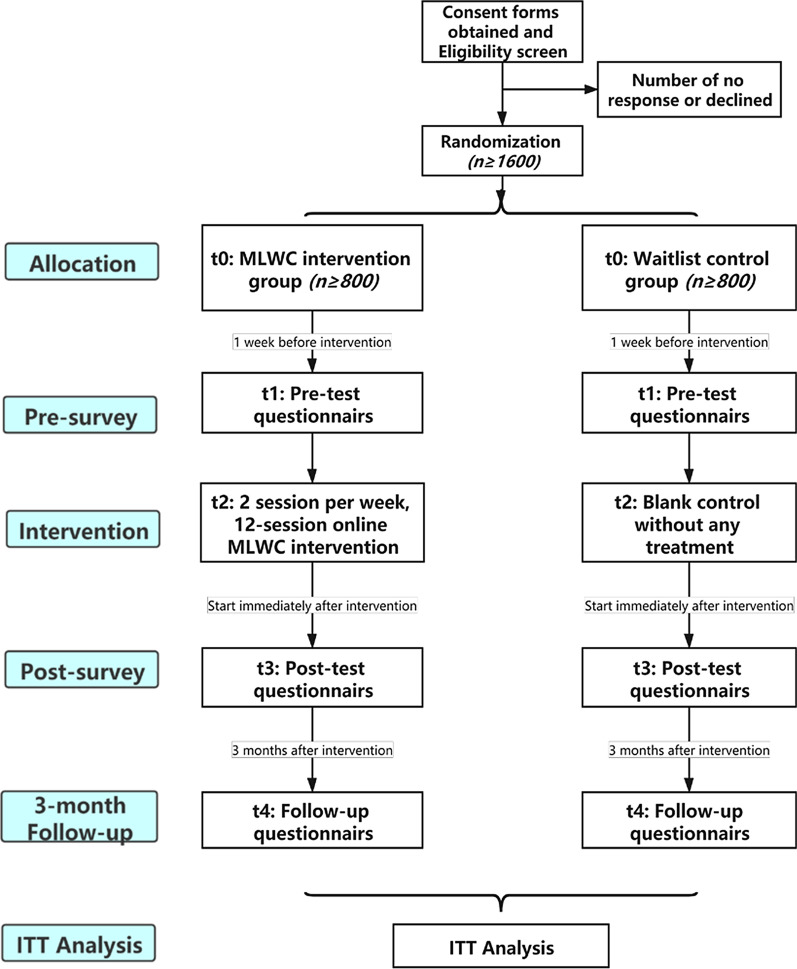

## Background

Coronavirus disease 2019 (COVID-19), the infection caused by severe acute respiratory syndrome coronavirus 2 (SARS-CoV-2), was first reported on December 31, 2019. On March 11, 2020, the World Health Organization (WHO) declared the COVID-19 as a pandemic due to its rapid global spreading. Globally, there have been 122 536 880 COVID-19 cases and 2 703 780 deaths, particularly among vulnerable populations such as patients with chronic diseases and the elderly [1, 2]. As of 24:00 pm on March 22, 2021, China alone has resulted in 102 566 cases and 4850 deaths due to COVID-19 [3]. It can be seen that the mortality rate of COVID-19 has been declining recently, which means that more and more patients have been released from the hospital after being cured. Therefore, the focus has now been shifted to taking measures to rehabilitate COVID-19 survivors, including the restoration of their physical functions and psychological development.

Evidence suggests that this disease has psychological consequences on healthcare workers, students, psychiatric patients and general workers, especially for COVID-19 patients [4–13]. Immediately after the outbreak, a considerable proportion of patients with COVID-19 reported post-traumatic stress disorder (PTSD) (more than 33.0%), depression (60.2%), and anxiety (55.3%) symptoms in Shanghai, China [9]. In addition, a survey conducted in March, 2020 showed that 45.9% of patients had symptoms of depression, 38.8% had anxiety and 54.1% had insomnia in Wuhan, China [10]. COVID-19 survivors in other countries have similar psychological status [11–13]. In Ecuador, one of the most affected countries by the COVID-19 pandemic in Latin America, of the 759 confirmed or suspected COVID-19 patients, 20.3% showed moderate to severe depressive symptoms, while 22.5% showed moderate to severe anxiety [11]. In addition, 22.6% and 16.3% of respondents reported clinically concerning levels of psychological impact in Vietnam and the Philippines, the Asian countries with similar cultural context as China [12, 13]. Over time, while the mental symptoms and quality of life of COVID-19 survivors might improve in a certain extent, they are still significantly worse when compared to the rest of the population who did not suffer from COVID-19. Therefore, the psychological impacts of COVID-19 should not be overlooked and should be investigated from the long-term public health perspectives.

In view of the current epidemic situation and the corresponding prevention and control requirements, internet-based interventions can provide an alternative to face-to-face therapy in enhancing mental health and quality of life for patients with COVID-19. Internet-based healthcare has enabled patients with a variety of geographical distance to receive the same therapy and get benefits from the same interventions, such as digital cognitive behavioural therapy for insomnia [14–16]. This form of psychotherapy can provide treatment and intervention efficiently to patients under the requirements of social distancing during the COVID-19 epidemic [14]. Mindfulness-based intervention (MBI) is an approach that focuses on the cultivation of conscious awareness, and orientation towards the present moment with curiosity and openness, which has been shown helpful in the treatment of mental health [17–20]. Unlike behavioral therapy, which involves relaxation techniques and changing the schedule of daily activities, mindfulness-based interventions help patients cope with anxiety and depression by enhancing their ability to manage stress and reducing negative coping behaviors, hence are more adaptive and persistent [20]. Especially when combined with the web-based technology, mindfulness-based intervention can benefit patients who are infected and treated in isolation wards, and those who are quarantined with no access to psychotherapy as well. [17, 20] An internet-based integrated intervention including some mindfulness skills on depression and anxiety symptoms in Chinese patients with COVID-19 has demonstrated that patients of the intervention group exhibited significantly decreased levels of depression and anxiety symptoms in comparison with those of the control group over a 2-week study period [18].

In the mainland of China, WeChat App and its small programs have dominated the browsing time of mobile phone users [21] Given the emerging need for psychological rehabilitation among COVID-19 survivors recently, this randomized controlled trial will be conducted in this population to examine the effectiveness and feasibility of a 6-week Mindful Living With Challenge (MLWC) intervention focusing on mental health and quality of life via WeChat. We hypothesized that the training could enhance the physical and mental health at post-program and 3-month follow-up.

## Methods/design

### Study design

**Fig. 1:**
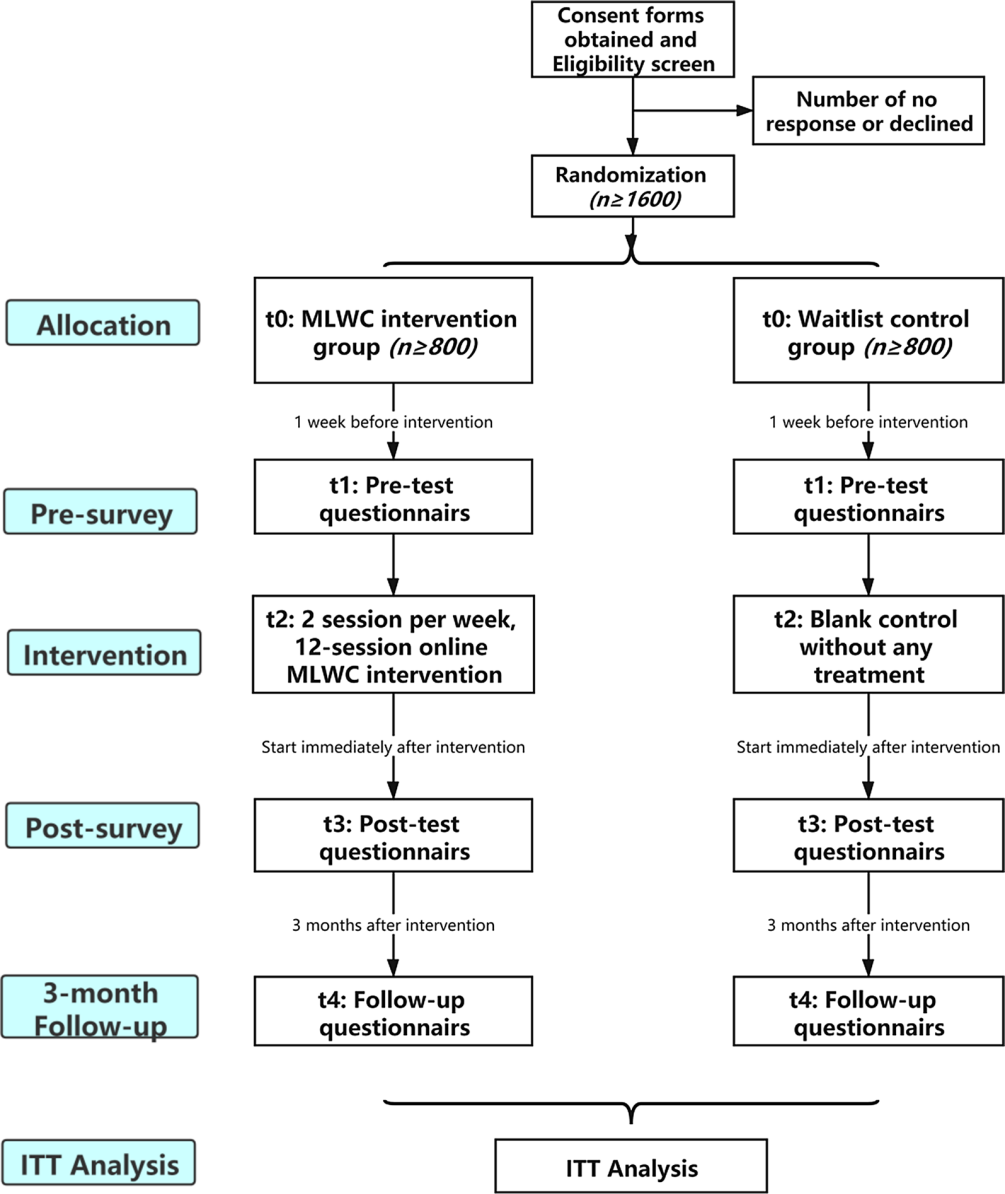
Flow chart of the protocol. MLWC: Mindful living with challenge; ITT: Intention-To-Treat

This is a prospective, randomized, parallel-group, double-blind, blank controlled trial involving an online 6-week MLWC intervention with a 3-month follow-up. More than 1600 COVID-19 survivors over 18 years old will be randomized to: (1) intervention group: this group will receive a baseline questionnaire followed by a 6-week web-based MLWC intervention, and two questionnaire surveys at post-program and 3 months after the intervention, or (2) waitlist control group: participants in this group will receive three questionnaire surveys conducted simultaneously with the intervention group, and start the same intervention after a 3-month waitlist period (Fig. 1). This study protocol is reported in accordance with SPIRIT reporting guidelines [22]

### Study objectives

The primary study objective of this study is to assess the efficacy of the MLWC intervention on PTSD, depression, and anxiety among COVID-19 survivors. The secondary study objective is to investigate the effects of the MLWC intervention on fatigue and sleep quality among COVID-19 survivors. The exploratory objective is to determine how mindfulness training affects mental health and quality of life among COVID-19 survivors and to evaluate the possible mediators and moderators (mindfulness, stillness, nonattachment, resilience and perceived social support) of the link between the MLWC intervention and target outcomes.

### Settings

The study will be carried out at the Hubei Provincial Hospital of Integrated Traditional Chinese and Western Medicine, Hubei Province, China. All researchers and support staff in this project will be trained based on the same protocol and are required to have an educational background in medicine or public health. Digital informed consent will be obtained from all subjects to ensure their voluntary participation. This study is planned to be performed in April 2021 and will last for about 4 months (The study has not been carried out before the submission).

### Sample size

A priori power analysis has been conducted on the statistical software G*Power 3.1.9.2 (Heinrich-Heine-Universität Düsseldorf). The trial between the MLWC intervention group and the control group will be tested superiority in promoting mental health status at a superiority margin of 10%. Thus, with a 1:1 allocation to each group and allowing for a 40% attrition rate, at least 1600 participants will be recruited (power = 0.9, α = 0.05, two-tail).

### Recruitment

Participants will be recruited through advertisements posted in hospitals and publicity by doctors in outpatient clinics or patient contact groups. Interested participants will be informed about the benefits and possible risks of participation in the study, including failure to achieve the expected intervention effects. Completion of electronic informed consent will be required before screening the eligibility of participating in the study.

Once the informed consent is obtained, a trained research staff will complete the eligibility checklists and record the candidates who fail to meet the inclusion criteria. Furthermore, a senior nurse will record the mobile phone number of participants to facilitate follow-up services. All of the above personal information will be kept confidential on an encrypted laptop and used for research purposes only. Table 1 shows schedule of enrolment, interventions and assessments.

**Table 1:** Schedule of enrolment, interventions, and assessments

Study period						
	Enrolment	Allocation	Post-allocation			Close-out
Time point	0	t_0_	t_1 baseline_	t_2 Intervention period_	t_3 Post-assessment_	t_4 Follow-up assessment_
*Enrolment*						
Eligibility screen	X					
Informed consent	X					
Allocation		X				
*Interventions*						
The Mindful Living with Challenge intervention group				X		
Waitlist control group				X		
*Assessments*						
Primary outcome measures						
Impact of Events Scale-Revised			X		X	X
Patient Health Questionnaire			X		X	X
Generalized Anxiety Disorder Questionnaire			X		X	X
Peace of Mind Scale			X		X	X
Secondary outcome measures						
Fatigue Scale-14			X		X	X
Pittsburgh Sleep Quality Index			X		X	X
The possible mediators and moderators						
Five Facets Mindfulness Questionnaire			X		X	X
The Nonattachment Scale			X		X	X
The Stillness Scale			X		X	X
The Resilience Style Questionnaire			X		X	X
The Social Support Scale			X		X	X

‘X’ represents the research procedure or content involved in each time point

### Eligibility criteria

#### Inclusion criteria

Participants must first agree to the electronic informed consent in order to participate in the study as required by the ethics committee. The participants have to meet the following criteria:

1. Over 18 and under 65 years old
2. Have a history of COVID-19 infection
3. Proficiency in Chinese
4. Be able to independently cooperate with doctors to complete various scale assessments
5. Have a mobile communication equipment such as a mobile phone, and a WeChat account.
6. Mobile equipment can access the internet at any time
7. Have not received medications for PTSD, depression, anxiety, fatigue, or sleep disorders within 1 month prior to enrollment in the study.

#### Exclusion criteria

Those who meet any of the below criteria will be excluded:

1. Have serious cognitive impairment.
2. Have serious heart, brain, lung, kidney, liver, and other medical diseases or tumors.
3. Difficult to cooperate with the questionnaire survey and intervention.

### Randomization and blinding

Participants who meet the eligibility criteria will be randomly assigned (in a 1:1 ratio) to the online MLWC intervention group or waitlist control group. They are supposed to provide informed consent by clicking the ‘I agree to join in the research’ button on the electronic details page of the study. After that, a Quick Response (QR) code will pop up on the page. By scanning this code, participants will be randomly assigned to the intervention or control group (in a 1:1 ratio) by computer-generated numbers. The trial will blind all people who might influence the outcome assessments, including the participants, data analysts, and outcome assessors. Before the trial, the principal investigator of the project will organize training on the randomization procedure and ensure that each research staff is clearly aware of their responsibilities.

### Intervention

#### The mindful living with challenge (MLWC) intervention

Participants in the intervention group are required to follow the developed WeChat small program by scanning the QR code, and then receive the online MLWC intervention. The MLWC intervention will be developed by two psychiatrists, and one of them has been certified in the Training of Mindfulness Facilitation program at the Mindful Awareness Research Center of University of California, Los Angeles (UCLA). The MLWC intervention adopts some elements from mindfulness-based stress reduction and Mindful Awareness Practices, and includes mindfulness meditation and mindful stretching as core components. A similar intervention has been successfully used on attention monitoring and acceptance in pregnant women in China [19]. Through a series of mindfulness training courses, participants are hypothesized to increase their ability to regulate their emotions and learn to observe the physical pain and psychological frustration caused by COVID-19 infection with equanimity. The purpose of the study is to help participants improve their skills in coping with psychological symptoms, improve their quality of life during recovery, and prepare to return to a normal life.

The MLWC intervention will involve internet-based mindfulness training conducted in six weekly courses of two sessions a week and 0.5 h per session, which focus on the four important parts of MBI: attention control, self-awareness, acceptance and discernment, and relating to difficult thoughts/emotions. Two new sessions of online lessons will be updated weekly in six weeks. The study participants are expected to spend an average of 1 h per week viewing each lesson, which consisted of a theoretical instrument, some meditation practices, and homework in the form of audio and video. Participants will be required to perform formal/informal mindfulness practice at home for 5 min daily initially, advancing to 20 min daily at the end of the intervention period, and to record or report their difficulties and progress during the practice. To enhance adherence, push notifications from the WeChat small program will be sent to participants daily to remind them about their weekly attendance and regular practice. See Table 2 for the context of the MLWC intervention.

**Table 2:** The content of mindful living with challenge (MLWC) intervention

Session	Theoretical instrument	Meditation practice	Homework
1	Explanation of the 5 facets of mindfulness (i.e. describing, acting with awareness, non-judging, and non-reacting), and of the importance of the mindfulness practices which is a means to attention control, how can we incorporate mindfulness into daily life, previous application and scientific findings of mindfulness based interventions	Mindful eating practice, mindful breathing meditation	Participants will be asked to conduct daily life mindfulness practices and mindful breathing meditation for 5–10 min daily for 7 days a week Participants will also be asked to record and provide feedback on any difficulties and their experience during the training
2	Explanation of the brain’s mode of action and being, thoughts/emotions/body feeling Instructing participants to talk to their bodies, relieve the pain and pressure in the healing process. Recognize the pain and try to persuade themselves to accept it, and realize self-awareness in the process	Mindful body scan practice, three-minute breathing space practice	Participants will be asked to conduct mindful body scan practice, three-minute breathing space practice, and filling in pleasant/unpleasant experiences calendar for 10–15 min daily for 7 days a week respectively Participants will also be asked to record and provide feedback on any difficulties and their experience during the training
3	Introduce the scientific understanding of sleep, sleep hygiene education, and seven attitudes of practicing mindfulness	Sitting meditation (mountain meditation and mindful sleep meditation)	Participants will be asked to conduct sitting meditation and mindfulness clock for 15 min daily for 7 days a week respectively Participants will also be asked to record and provide feedback on any difficulties and their experience during the training
4	Introduce mindful living with thoughts, using the **‘**STOP’ and ‘RAIN’ principle to dealing with a storm of thoughts and emotions	Mindful walking meditation, sitting meditation (lake meditation)	Participants will be asked to conduct mindfulness listening practice, mindfulness movement, and STOP/ RAIN practice for 15–20 min daily for 7 days a week respectively Participants will also be asked to record and provide feedback on any difficulties and their experience during the training
5	Introduce mindful movement for relaxing body and mind. Explanation of identification of avoidance response, allowing and letting it go	Mindful yoga practice	Participants will be asked to conduct sitting meditation for 15 min daily for 7 days a week and practice mindful yoga three times a day as appropriate Participants will also be asked to record and provide their feedback on any difficulties and their experience during the training
6	Introduce tired funnel and how to balance our daily life, mindful living with a challenge, and how to live a mindful life, live in the present	Sounding meditation; loving and kindness meditation	Participants will be asked to list nourishing/consuming activities and weave their own mindfulness parachute Participants will be encouraged to engage in various mindfulness practices on a daily basis in the future Participants will be asked to complete the post-program questionnaire and give feedback on the content, form, and organization of the intervention

#### Control group

Participants in the waitlist control group will receive the full 12-session online MLWC intervention upon completion of all treatment procedures by the intervention group after the 3-month follow-up survey.

## Measures

### Adherence

Participants will be informed of the importance of attending each session and required to complete the homework, as the effectiveness of the MLWC intervention on mental health and quality of life is dependent on adherence. Adherence to the intervention was examined through documentation of completed sessions. Firstly, the internet-based intervention platform can automatically record which participant has logged into each session and how much time they spend on mindfulness training per week. In addition, participants will be reminded to complete their homework after completion of each session. Moreover, the post-program questionnaire will be sent to the participants at the end of the intervention to ask about their adherence issues.

### Outcome measurements

All scales to be used in this study are validated Chinese version.

#### Primary outcome: PTSD, depression, anxiety and peace of mind

The psychological impact of COVID-19 will be assessed by the 22-item Impact of Event Scale-Revised (IES-R). IES-R is composed of three subscales and aims to measure the mean avoidance, intrusion, and hyperarousal (Cronbach's α = 0.80) [23–25]. Depression symptoms will be assessed by the 9-item Patient Health Questionnaire (PHQ-9) (Cronbach's α = 0.86) [26–28]. The level of general anxiety will be assessed by the 7-item Generalized Anxiety Disorder Questionnaire (Cronbach's α = 0.89) [29, 30]. Additionally, the 7-item Peace of Mind Scale developed by Lee et al. will be used to measure the peace of mind, one part of the well-being in the Chinese culture (Cronbach's α = 0.90) [31, 32].

#### Secondary outcomes: fatigue and sleep quality

Fatigue scale-14 (FS-14) assesses the patient's level of physical fatigue and mental fatigue (Cronbach's α = 0.81) [33, 34]. The 19-item Pittsburgh Sleep Quality Index (PSQI) assesses respondents’ sleep disturbances during the past month (Cronbach's α = 0.84). [35, 36] PSQI can be divided into seven component scores: subjective sleep quality, sleep latency, sleep duration, habitual sleep efficiency, sleep disturbances, use of medication, and daytime dysfunction [35].

#### The possible mediators and moderators: mindfulness, nonattachmet, stillness, resilience and perceived social support

The mindfulness level will be assessed by the 20-item short formed Five Facets of Mindfulness Questionnaire (FFMQ-SF). FFMQ-SF is a self-reported questionnaire with five mindfulness domains: observing, describing, acting with awareness, non-judgment, and non-reactivity (Cronbach's α = 0.86) [37, 38]. Nonattachment will be evaluated by the 7-Item Nonattachment Scale (NAS-7) (Cronbach's α = 0.82) [39–41]. To measure the extent to which participants experience stillness, the 11-item Stillness Scale will be used. It is developed and validated by one of the authors with three subscales: tranquility, concentration, and equanimity. (Cronbach’s α ranges from 0.72 to 0.75). In addition, participants' resilience will be measured by the 17-item Resilience Style Questionnaire (RSQ) (Cronbach’s α = 0.88) [42]. The level of emotional and material social support that participants receive will be measured with two items used in previous studies (Cronbach’s α = 0.68) [43].

### Data collection, management, and monitoring

Data will be collected at baseline (one week before the intervention), post-intervention (one week after the last session of MLWC), and at 3-month of follow-up. The questionnaire comprises structured and open-ended questions about mental health status, quality of life, and satisfaction with the program. Demographics will be collected at baseline, including age, employment status, education level, marital status, and other information.

Electronic questionnaires powered by the REDCap software will be used to collect data, hence there will be no formal data monitoring committee. Survey questions filled up by the participants will be automatically uploaded to the read-only web-based database to ensure the authenticity of the data. Moreover, our researchers will promptly check the collected data after each questionnaire survey.

### Statistical analysis

IMB SPSS Statistics 22.0 (IBM Corp., Armonk, NY, USA) will be used to process data and conduct a *t*-test, chi-square test, and correlation analysis. Analyses will be conducted based on an intention-to-treat approach, and two-sided with *P* < 0.05 will be considered statistically significant. Quantitative data accords with normal distribution will be described as mean ± standard deviation (*SD*), and qualitative data will be expressed as the number (percentage). Student’s *t*-test and the chi-square test will be employed to examine between-group differences in the baseline. The repeated measures ANOVA will be used to compare the scores for the six instruments, and the paired-samples *t*-test will be used to test for within-group differences. We will also conduct a correlation analysis to test the associations between variables in pre-program and post-program in each group. Structural Equation Model will be conducted to explore the interaction of the variables.

Furthermore, in order to elaborate on the meaning of the relationship between the mindfulness training and the outcome variables of mental state and quality of life among COVID-19 survivors, an analysis of mediator or moderator effects will be conducted and some more in-depth information about the research may be explained. We will use the hierarchical multiple regression analyses to test how or why some facets of mindfulness may take a role in our outcomes.

## Discussion

With the unprecedented global health challenge the COVID-19 pandemic has created, the secondary crisis of mental health problems occurred with the outbreak cannot be ignored in a very long period of time. As of March 22, 2021, there are 97 716 COVID-19 survivors cured and discharged from hospital in China, about one-third of them (i.e. 32 572 patients) may be suffering from mental illness as reported by the survey results in Wuhan, China [3, 10]. Prolonged exposure to stressful hospitalization, complex medical examination procedures, and worries about the difficulty of returning to normal life can lead to long-term psychological harm [44]. This will undoubtedly affect the normal operation of many families and bring huge health and economic pressure to the country. Therefore, appropriate innovative mental health support with social distancing merit is warranted to be implemented during the COVID-19 pandemic and its aftermath.

This article describes the design and content of a parallel-group randomized controlled trial (RCT). Our study is the first large sample size study to explore the effect and underlying mechanism of the online MLWC intervention aiming to improve mental health and quality of life among COVID-19 survivors in China. If the study demonstrates that the web-based MLWC is efficacious in such improvement, this user-friendly and convenient mode of mental health intervention would be valuable to treat psychological symptoms and improve their quality of life among COVID-19 rehabilitation patients. The intervention materials also have the potential to be utilized in other populations in various similar situations.

Although previous studies showed that face-to face mindfulness practices are effective to alleviate mental and physical symptoms including depression and sleeplessness [17–19, 45], the web-based mindfulness intervention were proved to have potential in improving the mental health of various study population with more feasibility and acceptability [46]. Currently, the WeChat App and its small programs have become an integral part of everyday life for the majority of the population in China [21]. To ensure the efficacy and the quality of the mindfulness intervention, the content of the intervention course will be developed by a team with clinical psychology background and delivered by a psychiatrist with mindfulness training qualification. Also, to enhance the adherence of the mindfulness-based intervention, professionals in information technology will design the specific small program for managing the questionnaire collection and distribution of the embedded video and audio courses during the study period.

The evaluation of the online MLWC intervention proposed in this study for the treatment of psychological symptoms among COVID-19 survivors is scarce in the international literature. The current study will use RCT, double-blind, evidence-based meditation design, and a longer period of intervention and follow-up, which may ensure more solid conclusions compared to similar studies [18, 47]. Moreover, since some studies have found that the mindfulness intervention did not affect all of the mindfulness facets [48, 49], we will explore 12 sessions of the MLWC intervention as intermediaries on the outcome measures, to determine which sessions have the greatest impact on the outcome, and provide more solid evidence for future psychological interventions. Furthermore, given the inexpensive and portable features of the online MLWC intervention, it can be more easily implemented in harder-to-reach but internet-accessible populations. However, it should be noted that, this study will mainly use questionnaires to measure psychiatric symptoms and do not make clinical diagnosis through structured clinical interview and functional neuroimaging, which may result in measurements that are inconsistent with clinical examinations [50, 51].

## Data Availability

The original data generated from this study and the analyzed results will be available from the corresponding author upon reasonable request.

## Abbreviations

COVID-19: Coronavirus disease 2019
MLWC: Mindful living with challenge
MBI: Mindfulness-based intervention
PTSD: Post-traumatic stress disorder
RCT: Randomized controlled trial
CONSORT: Consolidated Standards of Reporting Trials
IES-R: Impact of Events Scale-Revised
PHQ-9: Patient Health Questionnaire
GAD-7: Generalized Anxiety Disorder Questionnaire
PoM: Peace of Mind Scale
FS-14: Fatigue Scale-14
PSQI: Pittsburgh Sleep Quality Index
FFMQ: Five Facets Mindfulness Questionnaire
NAS-7: The Nonattachment Scale
RSQ: The Resilience Style Questionnaire
SD: Standard deviation
*P*: Probability

## Declarations

### Protocol amendments

Any modifications to the protocol which may impact on the conduct of the study will be approved by the Ethical Review Committee of Institute of Pathogen Biology prior to implementation. Other minor amendments will just be agreed upon by the cooperation team and documented in a memorandum.
